# Melatonin Improves Heat Tolerance in Kiwifruit Seedlings through Promoting Antioxidant Enzymatic Activity and Glutathione S-Transferase Transcription

**DOI:** 10.3390/molecules23030584

**Published:** 2018-03-06

**Authors:** Dong Liang, Fan Gao, Zhiyou Ni, Lijin Lin, Qunxian Deng, Yi Tang, Xun Wang, Xian Luo, Hui Xia

**Affiliations:** 1 College of Horticulture, Sichuan Agricultural University, Chengdu 611130, China; liangeast@sicau.edu.cn (D.L.); 18227551150@163.com (F.G.); nizhiyou123@sina.com (Z.N.); llj800924@163.com (L.L.); dqxlwj@sina.com (Q.D.); tangyisunguochao@sina.com (Y.T.); wangxun0104@hotmail.com (X.W.); lawxian@aliyun.com (X.L.); 2 Institute of Pomology and Olericulture, Sichuan Agricultural University, Chengdu 611130, China

**Keywords:** antioxidant enzymes, glutathione S-transferase, kiwifruit, melatonin, high temperature stress

## Abstract

Evidence exists to suggest that melatonin (MT) is important to abiotic stress tolerance in plants. Here, we investigated whether exogenous MT reduces heat damage on biological parameters and gene expression in kiwifruit (*Actinidia deliciosa*) seedlings. Pretreatment with MT alleviates heat-induced oxidative harm through reducing H_2_O_2_ content and increasing proline content. Moreover, MT application raised ascorbic acid (AsA) levels and the activity of antioxidant enzymes, including superoxide dismutase (SOD), catalase (CAT), and peroxidase (POD). We also observed elevation in the activity of enzymes related to the AsA-GSH cycle, such as ascorbate peroxidase (APX), monodehydroascorbate reductase (MDHAR), dehydroascorbate reductase (DHAR), and glutathione reductase (GR). Furthermore, MT application increased the expression of 28/31 glutathione S-transferase (GST) genes, reducing oxidative stress. These results clearly indicate that in kiwifruit, MT exerts a protective effect against heat-related damage through regulating antioxidant pathways.

## 1. Introduction

Temperatures 5 °C above optimal growing conditions induces heat shock or stress in plants, causing growth inhibition and crop failure [1,2]. These negative effects occur because cellular homeostasis is disrupted through mass formation of reactive oxygen species (ROS) in plant cells. These compounds include singlet oxygen (^1^O_2_), superoxide radical (O_2_^•−^), hydrogen peroxide (H_2_O_2_), and hydroxyl radical (OH^•^) are responsible for oxidative stress [3]. As a result, lipid peroxidation increases to cause oxidative stress, damaging membrane protein polymerization and cross-linking, as well as lowering membrane mobility, permeability, and thermal stability [4,5]. Like other aerobic organisms [6], plants have evolved defense systems that are well equipped with different antioxidant components to scavenge over-produced ROS, thus protecting plants from oxidative injury. An important aspect of these systems are antioxidant enzymes such as superoxide dismutase (SOD), catalase (CAT), peroxidase (POD), ascorbate peroxidase (APX), glutathione reductase (GR), monodehydroascorbate reductase (MDHAR), and dehydroascorbate (DHAR), as well as non-enzyme antioxidants such as ascorbic acid (AsA) and glutathione (GSH) [7,8]. In particular, glutathione S-transferases (EC 2.5.1.18) are a diverse, multifunctional group of stress-response enzymes, catalyzing GSH-dependent peroxidase reactions that scavenge toxic organic hydroperoxides. According to the genetic structure and protein homology, plant GSTs can be divided into 6 categories: Phi, Tau, Zeta, Lambda, Theta and Dehydroascorbate reductases (DHAR) [9,10].

Melatonin (MT) has received much recent attention in plant research because of its role as a growth regulator and a biostimulator for stress resistance [11]. The molecule enhances photosystem (PS) II activity [12]; alleviates growth inhibition and leaf senescence [13]; improves germination percentage [14], raise antioxidative enzymatic activity, antioxidant content [15,16]; and nitrogen metabolic enzyme activity [17]; as well as improve overall growth and rooting [18].

Kiwifruit (*Actinidia deliciosa*) is a perennial vine that is commercially cultivated in China, New Zealand, Chile, Japan, and Italy. Its heat-sensitivity is a major obstacle to crop productivity, however [19]. Long-term high temperatures cause flower and fruit dropping, quality deterioration, and storage decline [20]. Although a few studies on kiwifruit heat resistance are available [21,22], few researchers have examined how exogenous MT applications may improve antioxidation systems in kiwifruit seedlings under heat stress. Thus, the present study investigated the effectiveness of exogenous MT as an antioxidant-pathway regulator and an enhancer of heat-stress tolerance in kiwifruit.

## 2. Results

### 2.1. Seedling Morphology, H_2_O_2_ and Proline Content in Heat-Stressed Kiwifruit

**Figure 1:**
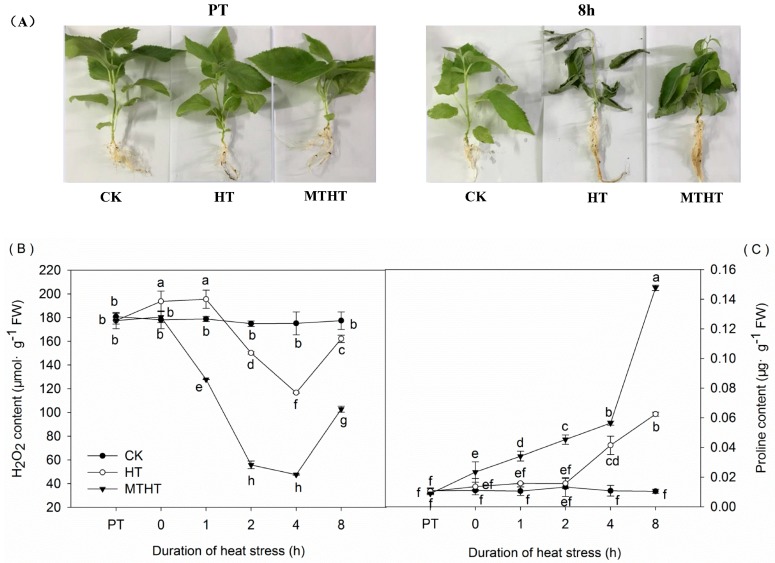
(**A**) Seedling morphology at PT (25 °C starting temperature) and after 8 h at 45 °C; (**B**) H_2_O_2_ content in leaves under heat stress; (**C**) Proline content in leaves under heat stress. CK, control; HT, high temperature treatment (45 °C); melatonin-pretreated high-temperature (MTHT), high temperature with melatonin pre-treatment. Data are means of three biological replicates (*n* = 3). Lowercase letters indicate significant differences (*p* < 0.05).

Before the experiment, seedlings were nearly identical across all three treatments (control [CK, 25 °C], high-temperature [HT, 45 °C], melatonin-pretreated high-temperature [MTHT, 45 °C) (Figure 1A). After 8 h treatment, HT seedlings exhibited dried leaves and water loss, whereas the MTHT group showed significantly fewer heat-stress symptoms (Figure 1A).

Under the first 2 h of HT, H_2_O_2_ content in kiwifruit seedling increased, but then rapidly decreased until 4 h of HT (Figure 1B). Notably, MTHT seedlings had significantly lower H_2_O_2_ levels than HT seedlings.

Proline prevents plant cell dehydration and protects cytoplasmic membrane integrity. In HT and MTHT kiwifruits, proline content gradually increased over time (Figure 1C), but by 8 h, MTHT seedlings contained 1.36 and 2.1 times more proline than CK and HT seedlings, respectively.

### 2.2. POD, CAT, and SOD Activities under Heat Stress

**Figure 2:**
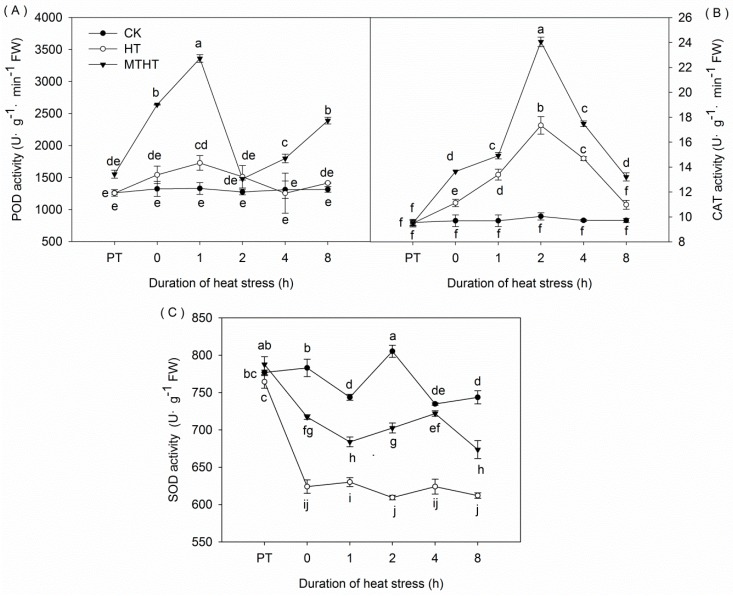
Antioxidant enzyme activity under heat stress. (**A**) Peroxidase (POD); (**B**) catalase (CAT); and (**C**) superoxide dismutase (SOD). Lowercase letters indicate significant differences (*p* < 0.05).

We observed a significant increase in POD activity that peaked at 1727.4 U·g^−1^·min^−1^ FW after 1 h of HT, before falling to CK levels after 4 h. Additionally, MT pretreatment dramatically increased POD activity, peaking after 1 h at 3360 U·g^−1^·min^−1^ FW, over two times greater than activity in HT leaves. After a drop at 2 h, POD activity in MTHT seedlings continued to increase (Figure 2A).

We recorded a persistent increase in CAT activity under heat stress, peaking at 2 h of treatment (HT: 17.35 U·g^−1^·min^−1^ FW, MTHT: 24.06 U·g^−1^·min^−1^ FW) (Figure 2B). Subsequently, CAT activity decreased in both MTHT and HT seedlings. Throughout the experiment, CAT activity was significantly higher in MTHT than in HT.

Heat stress immediately (at 0 h) caused a marked and rapid decrease of 20.31% in SOD activity among HT leaves compared with CK leaves (Figure 2C). This decline was halved in MTHT leaves.

### 2.3. Ascorbic Acid Content and AsA-GSH-Cycle Enzymatic Activity under Heat Stress

**Figure 3:**
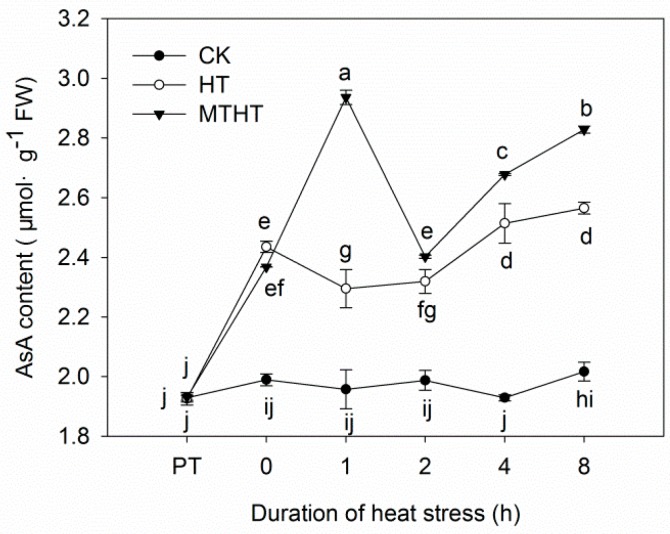
Ascorbic acid (AsA) content in kiwi leaves under heat stress. Lowercase letters indicate significant differences (*p* < 0.05).

Compared with the steady levels in CK, AsA content exhibited two peaks in both HT and MTHT (Figure 3). In MTHT, AsA peaked at 1 h, reaching a value that was 19.19% greater than corresponding values in HT. Overall, except at 0 h, MTHT seedlings had higher AsA content than HT seedlings.

**Figure 4:**
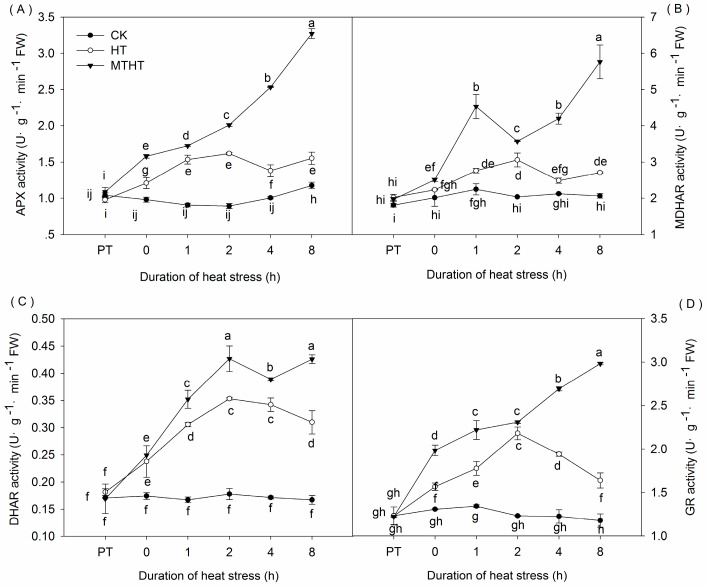
Activity of key enzymes from the AsA-GSH cycle in heat-stressed kiwi leaves. (**A**) Ascorbate peroxidase (APX); (**B**) monodehydroascorbate reductase (MDHAR); (**C**) dehydroascorbate (DHAR); and (**D**) glutathione reductase (GR). Lowercase letters indicate significant differences (*p* < 0.05).

Under heat stress, APX activity in HT increased steadily until 4 h, when activity began to fluctuate slightly. In contrast, APX activity in MTHT persistently increased over time, peaking at 8 h with 3.32 U·g^−1^·min^−1^ FW, twice as high as corresponding HT values (Figure 4A). Heat stress also increased MDHAR activity, most noticeably in MTHT. In these leaves, enzyme levels exhibited a wavelike pattern that peaked at 5.76 U·g^−1^·min^−1^ FW after 8 h, a 193.31% increase from levels at 0 h (Figure 4B). In both HT and MTHT, DHAR activity first rose steadily beyond CK levels, before decreasing after 2 h. At the 2 h peak level, DHAR activity in MTHT was 20.85% higher than in HT, and both values were higher than CK. (Figure 4C). Finally, GR activity in HT significantly increased, peaking at 2 h (2.18 U·g^−1^·min^−1^ FW) before decreasing. In contrast, GR activity rose continuously in MTHT, increasing by 143.89% at 8 h (Figure 4D).

### 2.4. Expression Profile of GST under Heat Stress

**Figure 5:**
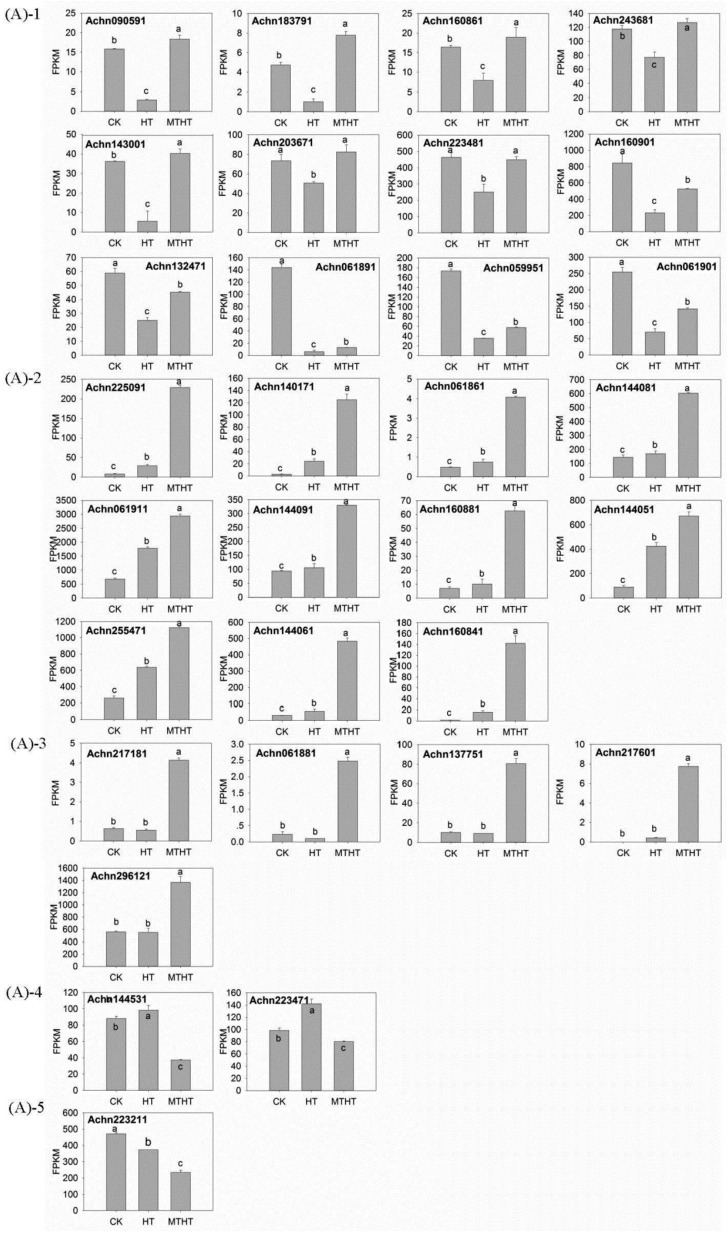

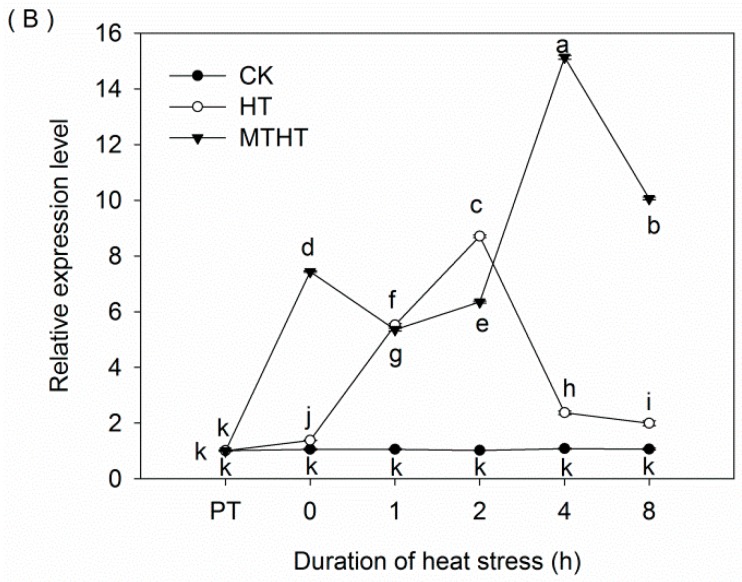
(**A**) Results of RNA-seq showing significant differences in *GST* gene expression between MTHT and HT; (**B**) Data from qRT-PCR showing *GST25* (ACHN160841 expression profile under heat stress. Lowercase letters indicate significant differences (*p* < 0.05).

Using RNA-seq data, we discovered *GST* gene expression patterns differed significantly between MTHT and HT (adjusted *p* < 0.05; Figure 5A). There were 25 Tau *GSTs*, 2 Lambda *GSTs*, 2 Theta *GSTs*, 1 Phi *GST* and 1 unknown *GST*. The 31 differentially expressed *GST* genes were classified in five groups based on their deviation from CK expression level. (1) *GST* was down-regulated in HT and up-regulated in MTHT (12 transcripts); (2) *GST* was up-regulated in both HT and MTHT (11 transcripts); (3) *GST* remained unchanged in HT and up-regulated in MTHT (5 transcripts); (4) *GST* was up-regulated in HT and down-regulated in MTHT (2 transcripts); (5) Finally, *GST* was down-regulated in HT and MTHT (1 transcript). Overall, MT significantly up-regulated 28 *GST* genes, and only down-regulated 3.

Next, we selected *GST25* (ACHN160841) in Group 2 for quantitative real-time PCR (qRT-PCR) analysis, to understand how *GST* expression changed over time (Figure 5B). Compared with CK, *GST* expression in HT first increased and then decreased. Additionally, *GST* gene expression in MTHT increased by 439.36%, 539.09%, and 495.04% at 0, 4, and 8 h, respectively, compared with HT (Figure 5).

## 3. Discussion

Melatonin is a well-documented antioxidant in plants that is critical to alleviating environmental stress [23,24,25,26]. Here, we observed that one key way exogenous MT increased kiwifruit heat resistance was through decreasing H_2_O_2_ content, in accordance with other work on *Malus*, *Cynodon dactylon*, and cucumber [27,28,29]. The mechanism underlying H_2_O_2_ reduction is likely the fact that MT acts as an electron donor [24]. Additionally, SOD catalyzes the removal of O_2_^•−^ by dismutating it into O_2_ and H_2_O_2_ [30]; CAT and POD are involved in scavenging H_2_O_2_ to H_2_O and O_2_ [31]. In our study, we found H_2_O_2_ content in HT is lower than in CK, which may be because of the SOD reduction activity was not enough to counteract the occurring oxidative load. We, observed that under heat stress, MT enhanced the activity of major antioxidant enzymes (SOD, CAT, POD), possibly through upregulation of relevant genes. These results are similar to findings in cold-stressed cucumber and pepper seeds, showing that MT increased SOD activity through various physiological and molecular mechanisms in response to decreased H_2_O_2_ [15,16]. Likewise, our data correspond to results of MT treatment on stressed tea [32] and wheat [33]. Furthermore, as observed in wheat seedling [34,35], we found that heat stress increased proline content in kiwi leaves, perhaps because stress abolished feedback inhibition in the proline biosynthetic pathway [36]. Furthermore, MT pretreatment magnified this increase, as reported in cherry [37] and tomatoes [38]. These patterns may be attributable to the maintenance of low cell osmotic potential and reduced water loss through MT-induced proline accumulation, allowing improved adaption to a hot environment [39,40].

In our study, exogenous MT increased AsA content through elevating MDHAR and DHAR activity. Moreover, MT treatment increased GR activity more than it increased DHAR activity. These compounds are all part of the AsA-GSH cycle, an important antioxidant pathway that generates the small-molecule, non-enzymatic antioxidants AsA and GSH [41]. Fluctuation in AsA content is dependent on APX, MDHAR, and DHAR activities, with the latter two responsible for recycling AsA. Additionally, DHAR oxidizes GSH to GSSG during ROS scavenging, while GR recycles GSH. Our data thus suggest that exogenous MT is important to AsA and GSH biosynthesis/regeneration. Overall, we demonstrated that enhancing the AsA-GSH cycle is another way exogenous MT can protect plant tissues from oxidative damage [42,43].

Finally, we discovered 31 differentially expressed *GST* genes between MTHT and HT kiwifruit. They had five expression patterns which may be due to different functions of the gene family [9,44]. Glutathione S-transferases are critical to plant development and stress response through their scavenging of peroxides and other electrophiles [45,46,47,48,49,50]. Indeed, *GST* over-expression improves abiotic stress tolerance in tobacco and *Arabidopsis* [51,52]. The fact that we observed more up-regulated than down-regulated genes suggest that MT may dramatically decrease free-radical production and improve plant heat tolerance through elevating *GST* transcript abundance [53,54]. In general, our results proved that MT can improve the heat tolerance of heat-sensitive plants, moreover provided a way to make heat-sensitive plants grow better under heat stress.

## 4. Materials and Methods

### 4.1. Plant Materials and Treatment

Kiwifruit seeds were first disinfected for 5 min using 5% sodium hypochlorite and rinsed with distilled water. Cleaned seeds were grown at 4 °C and 60–70% relative humidity for 60 days. After a week-long poikilothermic treatment at 4 °C for 10 h and 25 °C for 14 h, germinated seeds were planted in plastic pots (diameter: 18 cm; height: 23 cm) filled with sand. They were then moved to a phytotron at Sichuan Agricultural University, Chengdu, China (30°42′ N, 103°51′ E), under conditions of 25/20 °C (day/night) and a 12/12 h (day/night) photoperiod. At the two-true-leaf stage, seedlings were watered in 2 days intervals with 1/2 Hoagland’s nutrient solution (pH adjusted to 6.5 ± 0.1 with diluted HCl or NaOH).

Treatments began at the 10-true-leaf stage. First, CK plants were maintained at 25 °C throughout the entire experiment. Second, HT seedlings were transferred to an incubator that increased from 25 °C to 45 °C across 2 h, and then maintained at the latter temperature for 8 h. Third, MTHT seedlings were pretreated 5 times with 200 µM MT solution, every two days, and then subjected to the same conditions as HT plants. Each treatment was performed in triplicate. The moment when the incubator temperature at 25 °C, was designed as PT; the moment when the incubator temperature just reached at 45 °C, was designed as 0 h. Five to eight middle leaves per plant were sampled at PT, 0, 1, 2, 4, and 8 h. All collected tissues were immediately frozen in liquid nitrogen and stored at −80 °C.

### 4.2. Assays of H_2_O_2_ Content and Antioxidant Enzyme Activity

Determination of H_2_O_2_ and proline levels followed previously described methods [55,56].

The photochemical reduction of NBT [57] was used to assay SOD activity. The guaiacol colorimetric method [58] was employed for measuring POD activity. Finally, CAT activity was calculated as the decline in A240 [59].

### 4.3. Extraction and Assay of AsA Content and AsA-GSH Cycle Enzymes

Ascorbic acid content was measured following existing methods [60]. Briefly, leaves (0.3 g) were ground in a prechilled mortar, then homogenized in 5 mL of ice-cold 6% (*v*/*v*) trichloroacetic acid (TCA) and 1 mM EDTA- Na_2_ solution. Crude extract was centrifuged at 2 °C and 12,000 *g* for 10 min; the supernatant was collected for analysis. We neutralized 50 μL extract with 250 μL 10% (*w*/*v*) TCA, 200 μL 42% H_3_PO_4_, and 200 μL 2% (*w*/*v*) 2,2-Dipyridyl (C_10_H_8_N_2_). The assay was performed using a spectrophotometer at 525 nm in 200 mm sodium phosphate buffer (pH 7.4), both before and after a 60 min incubation at 42 °C with 100 μL of 3% (*w*/*v*) FeCl_3_.

To determine the activity of enzymes involved in the AsA-GSH cycle, leaves (0.5 g) were ground in a chilled mortar with 4% (*w*/*v*) polyvinylpolypyrrolidone, then homogenized with 8 mL of 50 mM potassium phosphate buffer (pH 7.5) containing 1 mM EDTA-Na_2_ and 0.3% Triton X-100. The activity of APX was determined via monitoring absorbance decreases at 290 nm as reduced H_2_O_2_ was oxidized [61]. Similarly, MDHAR activity was assayed through monitoring absorbance decreases at 340 nm as NADH was oxidized [62]. Next, DHAR activity was determined through absorbance increases at 265 nm due to dehydroascorbate (DHA) formation [61]. Finally, GR activity was assayed through absorbance decreases at 340 nm from NADPH oxidation [63].

### 4.4. Quantitative Real-Time PCR for Profiling GST Expression

Total RNA was extracted from frozen fresh leaves using a modified CTAB method and treated with RNase-free DNase I (Takara, Dalian, China) to remove genomic DNA contamination. The NanoPhotometer^®^ spectrophotometer (IMPLEN, Westlake Village, CA, USA) was used to check RNA purity. Quantitative real-time PCR was used to determine one selected *GST* gene. These reactions were performed on the CFX96 Real-Time System C1000 Thermal Cycler (Bio-RAD, Hercules, CA, USA), following manufacturer protocol in a SYBR Premix Ex Taq kit (TaKaRa, Dalian, China), and analyzed with 2^−ΔΔCT^. Relative gene expression was normalized with kiwifruit *Actin1* and *Actin2* [64]. Table 1 contains the primer sequences used for PCR. Three replicates were performed for three separate RNA extracts from three samples.

### 4.5. Expression Analysis of GST Genes Based on Transcriptome Data

Six cDNA libraries were constructed for three 8 h treatments (CK, HT, and MTHT), each with two biological replicates. Sequencing libraries were generated using the NEBNext Ultra RNA Library Prep Kit for Illumina (NEB, Ipswich, MA, USA) and sequenced on an Illumina Hiseq 2000 platform. Paired-end reads (150 bp) were generated by Novogene (Beijing, China).

Reads numbers mapped to each gene were counted in HTSeq version 0.6.1. The FPKM per gene was calculated based on gene length and read count. Differential expression analysis of two biological replicates was performed in R with the DESeq package (version 1.18.0, European Molecular Biology Laboratory, Heidelberg, Germany). The resultant P-values were adjusted using the Benjamini-Hochberg procedure for controlling false discovery rate [65]. Adjusted *p* < 0.05 was considered significant differential expression. 

## Figures and Tables

**Table 1:** qRT-PCR primer sequences.

Gene Locus	Forward Primer	Reverse Primer
ACHN160841	GGTGTTGATACATAACGGAAAG	TGGACAATGATGAGGGACT
*Actin1*	GCAGGAATCCATGAGACTACC	GTCTGCGATACCAGGGAACAT
